# Nitroglycerine in Angina Pectoris

**Published:** 1898-02

**Authors:** 


**Nitroglycerine in Angina Pectoris.**—The most efficacious form of administering nitroglycerine in angina pectoris:

| | | |
|---|---|---|
| ℞ | Nitroglycerine | gr. iii |
| | Tinct. capsici | ℨ ss |
| | Spts. rectificat | ℨ iii |
| | Aquæ menth. pip | ℨ iii |

M. Sig.: Two to ten drops.

In one minute the action of the drug is manifest, and in scarcely three minutes the pain is entirely done away with. As the patient grows accustomed to the dose it must be increased, and if this be done carefully there is no great danger to be anticipated.—*Dr. Schoot, in Wiener Klinische Rundschau.*

